# Development and validation of the civil aviation occupational gender stereotype scale and its impact on professional identity and self-efficacy among chinese civil aviation majors

**DOI:** 10.3389/fpsyg.2026.1718534

**Published:** 2026-09-30

**Authors:** Lingfeng Guo, Mengran Yang, Xiuyi Li, Xuelu Li, Qi Zhu, Junjie Zhang, Na Xie, Zhiqi Yin, Yanzhe Zhang, Xiaoqing Li, Yao Zhang, Yuchuan Luo, Zhi Xu

**Affiliations:** 1 Flight Technology College, Civil Aviation Flight University of China, Guanghan, China; 2 Sichuan Provincial Engineering Research Center of Domestic Civil Aircraft Flight and Operation Support, Guanghan, China; 3 Flight Training Center of Civil Aviation Flight University of China, Guanghan, Sichuan, China; 4 College of Humanities and Arts, Civil Aviation Flight University of China, Guanghan, Sichuan, China; 5 College of Aviation Engineering, Civil Aviation Flight University of China, Guanghan, Sichuan, China; 6 College of Aviation Physical Education, Civil Aviation Flight University of China, Guanghan, Sichuan, China

**Keywords:** civil aviation majors, civil aviation occupational gender stereotypes, professional identity, scale development, self-efficacy

## Abstract

**Introduction:**

This research aimed to develop and validate the Civil Aviation Occupational Gender Stereotype Scale (CA-OGSS) and investigate its associations with professional identity and self-efficacy among Chinese civil aviation majors, thereby providing an empirical foundation for fostering gender equality in the field.

**Methods:**

In Study 1, an initial 15-item pool was generated and refined through a literature review, interviews, and expert evaluation; its factor structure and reliability were examined using exploratory factor analysis (Sample 1, *n* = 707) and confirmatory factor analysis (Sample 2, *n* = 1,465). In Study 2, the finalized 10-item CA-OGSS, alongside established measures of professional identity and self-efficacy, was administered among 830 civil aviation majors to test a moderated mediation model.

**Results:**

The CA-OGSS demonstrated a stable two-factor structure, explaining 73.12% of the total variance in EFA. The final CFA model showed acceptable fit comparative fit index = 0.995; Tucker–Lewis index = 0.989; root mean square error of approximation = 0.072 [90% CI: 0.062, 0.084]; standardized root mean squared residual = 0.013), although the χ^2^/*df* value (6.77) exceeded the conventional cutoff. Reliability was acceptable. Path analysis revealed that gender stereotypes were associated with self-efficacy through professional identity, and this indirect association was moderated by gender. Simple slope analysis suggested that the association was negative among women but positive among men; the indirect association via professional identity was stronger among men than among women.

**Discussion:**

The findings indicate that CA-OGSS is a valid and reliable tool and underscore the importance of gender stereotypes in relation to the outcomes of civil aviation students, suggesting that future interventions to promote gender equality must account for these differential effects across genders.

## Introduction

Amid China’s ongoing efforts to advance gender equality as a fundamental national policy, eliminating workplace gender discrimination and dismantling occupational gender stereotypes have emerged as pressing societal imperatives (National Working Committee on Children and Women under State Council, 2025). The term “stereotype” was coined by Lippmann (Lippmann, 1922), who conceptualized it as a cognitive schema that simplifies information processing and thereby enables individuals to rapidly interpret and categorize complex social phenomena. However, in practice, stereotypes typically foster preconceived and biased judgments regarding their targets, which are generally unjust and may cause systematic errors (Devine et al., 1991). This automatic cognitive process operates at both conscious (explicit) and unconscious (implicit) levels, shaped by numerous factors, including historical context, sociocultural norms, implicit attitudes, and self-esteem (Liu et al., 1998).

Since ancient times, Confucian culture has profoundly influenced Chinese society. Feudal ethical codes (封建礼教), rooted in Confucianism, functioned as a system of rituals and moral principles imposed by the ruling class to regulate social behavior. These codes established hierarchical norms governing relationships between ruler and subject, father and son, husband and wife, older and younger siblings, and friends (Li and Chen, 2013). In this framework, gender was a foundational organizing principle. Doctrines such as the “Three Cardinal Guides and Five Constant Virtues [三纲五常]” and “Three Obediences and Four Virtues [三从四德]” institutionalized men’s supremacy and women’s subordination, relegating the latter to the status of the former’s dependents – “obeying fathers before marriage and husbands after marriage” – while systematically excluding them from public participation. This cultural paradigm cemented a rigid gender division of labor, epitomized by the adage “men rule outside, women rule inside [男主外, 女主内]” (Zhang, 2010). Over centuries, these norms became deeply entrenched and extended into occupational spheres, wherein they were further institutionalized and internalized as collective social cognition. Similarly, beyond the Chinese cultural context, the impact of gender stereotypes on career development has shown cross-cultural consistency. For example, research in the hotel industry found significant differences between male and female entrepreneurs in their perceptions of the factors constituting management (Moraleda et al., 2014); a review in the management field revealed that mechanisms such as “Think Manager–Think Male” hinder women’s career advancement (Tabassum and Nayak, 2021); and research in STEM fields has also shown that masculine field culture can weaken women’s willingness to participate (Cheryan et al., 2017).

In this cultural context, gender stereotypes – as a specific form of stereotypes – continue to influence contemporary societal evaluations of men and women across behavior, personality traits, and abilities (Ma, 2000; Qian et al., 1999). Qin and Yu (2001) found that the most important personality traits for men were creativity, humor, self-reliance, optimism, and competence, whereas those for women were self-reliance, kindness, gentleness, and tenderness. These lexical patterns reflect the divergent standards applied to men and women. Such evaluative criteria implicitly mirror broader sociocultural beliefs that associate women with high warmth and men with high competence (Kahalon et al., 2019).

This aligns with the stereotype content model (SCM), developed by Fiske et al. (Fiske et al., 2002), which posits that stereotypes across cultures are organized along the following two core dimensions: competence and warmth. Social groups are positioned in distinct quadrants of a two-dimensional space based on their perceived levels of these traits. Cross-cultural studies employing SCM have revealed the prevalence of mixed stereotypes, wherein perceived competence and warmth usually exhibit a negative correlation. For example, wealthy individuals are frequently stereotyped as high in competence but low in warmth, whereas homemakers are generally perceived as high in warmth but low in competence (Cuddy et al., 2009; Tchounwou et al., 2021). Notably, gender represents one of the most typical applications of the SCM. Within this framework, men are typically positioned as “high in competence, low in warmth,” whereas women are positioned as “high in warmth, low in competence” (Kahalon et al., 2019; Ma, 2000; Qian et al., 1999; Qin and Yu, 2001). This gendered competence–warmth perception is not confined to evaluations of individuals; it extends into the occupational domain, giving rise to a gendered division of labor in which men are considered suited to technical occupations and women to service-oriented ones (Eagly and Karau, 2002; Hong, 2024; Yu, 2003). Specifically, technical occupations (e.g., engineers, pilots, programmers) are perceived as requiring “rationality, precision, and stress resistance” – traits associated with high competence and masculinity – whereas service occupations (e.g., nurses, teachers, flight attendants) are perceived as requiring “attentiveness, warmth, and patience” – traits associated with high warmth and femininity (Forsman and Barth, 2017; Hu et al., 2004). Gender stereotypes and occupational stereotypes are thus intertwined, jointly shaping societal perceptions of the gendered nature of different professions.

However, this gendered division of labor is by no means value-neutral; it carries systematic and asymmetrical consequences. Even ostensibly “positive” gender stereotypes – such as ascribing “high warmth” to women – may perpetuate structural oppression and discrimination. On the one hand, such stereotypes can reinforce beliefs regarding gendered abilities and interests, thereby restricting the participation of men and women across diverse professions (Eagly and Karau, 2002). For instance, in STEM fields (Science, Technology, Engineering, and Mathematics), which are stereotypically associated with high competence, women’s participation remains significantly lower than men’s, resulting in the systematic underestimation and restriction of their potential and achievements (Hong, 2024). On the other hand, stereotypes typically link high-competence professions with men while associating high-warmth service occupations with women (Yu, 2003). However, the social valuation of “female warmth” and “male competence” is asymmetrical; that is, traits associated with women are often culturally devalued (Jackson and Bussey, 2020; Jackson et al., 2021). Specifically, occupational groups perceived as high in competence (such as white-collar employees in multinational companies, businesspeople, and lawyers) receive higher social prestige, whereas those associated with high warmth (such as hotel staff or community officers) are typically viewed less favorably (Hu et al., 2004). This differential evaluation of masculine and feminine traits likewise influences career preferences: Women exhibit comparable interest in both female-typed and male-typed occupations, whereas men express significantly lower interest in female-typed jobs (Forsman and Barth, 2017). As mentioned earlier, even “positive” stereotypes may perpetuate benevolent sexism. Unlike hostile sexism (e.g., “women are incompetent at work”), benevolent sexism (e.g., “women ought to be protected”) limits women’s career advancement in traditionally men-dominated fields and confines them to socio-emotional roles, steering them toward “stable and easy” traditionally female occupations, thereby reinforcing gender segregation in the labor market (Glick and Fiske, 2018). For example, Song and Chang (2025) study of 410 Chinese women across various industries found that benevolent sexism reduces self-esteem and elevates burnout, adversely affecting their career development.

Thus, gender stereotypes are closely linked to and interact dynamically with occupational stereotypes. This entails pre-judging the “appropriate” gender for certain occupations, whereby traits and abilities embedded in gender stereotypes are aligned with those considered necessary for specific jobs (Krefting et al., 1978; Lian et al., 2018). Consequently, these stereotypical associations significantly influence individuals’ career choices and social development.

Although the *China Women’s Development Outline* (State Council, 2021) explicitly calls for eliminating gender discrimination in employment, gender stereotypes in the sociocultural context of mainland China continue to exert a persistent influence across various professional fields. In the nursing sector, men students commonly exhibit both implicit and explicit occupational gender stereotypes, relatively low professional identity, and a negative correlation between these stereotypes and professional identity (Wang, 2022). Likewise, compared to women students, men students majoring in early childhood education demonstrate significantly lower levels of professional identity, professional efficacy, and academic achievement, while reporting significantly higher levels of occupational gender stereotyping (Xin, 2021). Such entrenched associations between gender and certain professions may cause individuals whose gender is underrepresented in a particular field to develop a weaker professional identity, which, in turn, can diminish self-efficacy.

The civil aviation industry is a strategically important sector for China’s economic and social development (Civil Aviation Administration of China, 2015). However, occupational gender stereotypes in this industry may significantly impede the effective alignment of professional talent with job roles. In mainland China’s civil aviation sector, men are predominantly employed in technically demanding positions, such as pilots, air traffic controllers, and aircraft maintenance engineers, whereas women are largely concentrated in service-oriented roles, such as flight attendants and ground staff. This gendered division of labor constrains women’s career advancement in the industry and fosters social disapproval or skepticism toward men who pursue traditionally feminized occupations. Such occupational gender stereotypes reinforce the perception that technical roles necessitate traits such as “rationality, precision, and stress resistance,” which are stereotypically associated with masculinity, whereas service roles are closely linked to “attentiveness, warmth, and patience,” which are conventionally feminized. The resulting negative impacts are both profound and bidirectional. For women, this stereotyping limits opportunities for upward mobility. Despite possessing strong technical competencies and professional ambition, women may encounter implicit biases for deviating from expected gender norms (Murphy et al., 2007), facing invisible barriers in recruitment and promotion that prevent them from entering core technical fields, such as piloting and air traffic control. This predicament constrains individual career potential and artificially diminishes the talent pool available to the industry as a whole. For men, engaging in traditionally feminine roles involves navigating identity conflicts and social stigma. Ferguson and Ayuttacorn’s study of male flight attendants in North America and Thailand revealed that airlines reinforce a binary narrative equating “pilots with masculinity” and “flight attendants with femininity.” Accordingly, men flight attendants typically experience a conflict between their professional identity and societal expectations of masculinity, resulting in them performing exaggerated masculine traits to counter prejudice (Ferguson and Ayuttacorn, 2019). This suggests that men in service-oriented aviation roles also expend additional emotional and psychological effort to cope with stereotyping, potentially undermining their job satisfaction and career development.

Based on Social Identity Theory, Tajfel and Turner proposed that individuals define themselves through group affiliation, and professional identity is a specific manifestation of this process in the occupational domain (Tajfel and Turner, 2004). Professional identity refers to the unity of cognition, emotion, and value evaluation regarding one’s occupation, encompassing dimensions such as professional cognition, professional emotion, professional behavioral tendency, and professional fit (Qin, 2009). Studies have shown that a high level of professional identity can enhance an individual’s self-efficacy and thereby alleviate career burnout. Self-efficacy, proposed by Bandura in social cognitive theory, is defined as an individual’s belief in their ability to organize and execute specific actions to achieve expected goals (Bandura, 1997). In the field of career development, self-efficacy has been demonstrated to predict individuals’ career life cycle (Lent et al., 1986). Based on the established positive relationship between professional identity and self-efficacy (Su, 2024), as well as evidence of the negative impact of gender stereotypes in fields such as nursing and STEM (Clark et al., 2021; Hong, 2024; Wang and Yu, 2019; Xin, 2021), we speculate that gender stereotypes in the civil aviation field may adversely affect individuals’ self-efficacy by weakening their professional identity. However, this relational pathway among the three variables has not yet been empirically tested.

Currently, research examining occupational gender stereotypes in the civil aviation industry from a psychometric perspective remains limited. Existing studies have predominantly employed interview-based methodologies and focused largely on pilots and flight attendants (Mallory and Casebolt, 2023; Nussrat, 2024). However, other critical roles – such as aircraft maintenance engineers and aviation security personnel – have received significantly less attention, leaving a considerable gap in our understanding of gender stereotypes across these essential positions. This oversight is particularly notable because the core principle of crew resource management emphasizes that flight safety and operational efficiency depend on the seamless coordination of all operational units – including the cockpit, cabin crew, and ground support – with each individual contributing indispensable professional value. How gender stereotypes manifest across different aviation professions and how students perceive and form attitudes toward the alignment between gender and specific roles in civil aviation remain underexplored. The mechanisms whereby these perceptions influence career choices and professional development are largely unknown. Simultaneously, a growing number of aviation students are pursuing specialties traditionally dominated by the opposite gender. Nevertheless, persistent occupational gender stereotypes continue to influence individuals’ career decisions as well as constrain gender diversity in the industry.

Therefore, investigating the structure and impact of occupational gender stereotypes in civil aviation is significant. Theoretically, such an investigation could contribute to the development of stereotype-related frameworks tailored to aviation contexts, and provide preliminary insights into how occupational gender stereotypes relate to individual development (i.e., professional identity and self-efficacy). Practically, it could provide empirical evidence to support efforts aimed at dismantling gender-based occupational segregation and promoting more equitable and efficient allocation of talent in the sector. Specifically, Study 1 developed and validated a domain-specific measurement tool, the CA-OGSS, to assess the structure of occupational gender stereotypes in civil aviation within the Chinese cultural context. Study 2 then examined the relationships between these stereotypes and two career-related outcomes – professional identity and self-efficacy – among Chinese civil aviation students, and further explored the potential moderating role of gender in these associations. This study was approved by the Ethics Committee of Civil Aviation Flight University of China (Ethics Committee Reference Number: CAFUC-2025审2号).

## Study 1: structure of the civil aviation occupational gender stereotype scale (CA-OGSS)

### Study 1a: item pool development

#### Methods

***Participants.*** We recruited participants from civil aviation universities. Eleven students representing key civil aviation majors – including two flight technology (pilot training) students, two air traffic control trainees, two aviation security students, three aircraft maintenance technicians, and two flight attendants – participated in the interviews (first stage). Furthermore, 19 students participated in the pre-test.

***Procedure.*** The item pool development involved the following stages: a literature review, interviews with students with civil aviation majors, an expert review and research group discussion, a pre-test, and a final expert review and research group discussion.

#### Results

***Literature review.*** The SCM model holds that technical professions prioritize competence, whereas service-oriented occupations emphasize warmth. Men-dominated fields are typically associated with strong technical capabilities, whereas women-dominated professions are generally linked to high levels of warmth and service orientation. Based on the SCM model, we designed interview protocols targeting the competence and warmth dimensions of occupational gender stereotypes. Additionally, we collected existing stereotype scales for other profession types from 31 prior studies. These scales, comprising 86 items, were systematically organized according to the two core SCM dimensions – namely, warmth and competence – with their respective items categorized accordingly.

***Interviews with students with civil aviation majors.*** The interviews focused on participants’ perceptions of gender stereotypes in civil aviation professions, the pressure they experienced owing to these stereotypes, and the potential impact on future employment. We recruited civil aviation students for online semi-structured interviews (approximately 20 min each). Sampling ceased after theoretical saturation was reached, yielding a total of 11 interview transcripts. Prior to the interviews, two psychology students who would conduct the interviews and later be responsible for transcription and coding received one session of offline training. The interview data were processed using NVivo, and a three-level coding procedure (open coding, axial coding, and selective coding) was performed based on procedural grounded theory. During the open coding stage, each transcript was independently coded back-to-back by two coders. When disagreements arose, the two coders responsible for that transcript compared their coding rationales item by item and discussed them thoroughly in light of the original interview context. If disagreements persisted, an expert in educational psychology and psychometrics was invited to adjudicate. Next, one coder conducted axial coding on the basis of the open codes to identify intrinsic relationships among concepts and to distill more general categories and dimensions. Finally, selective coding was performed to further integrate the relationships among categories and to extract core categories that could encompass all the data.

Through constant comparison and integration of concepts with shared meanings, and after discarding concepts that were clearly irrelevant to the research questions or appeared with excessively low frequency, 121 first-level open coding concepts were identified across the 11 interviews (e.g., employment discrimination and prejudice, gender–occupation fit, work competence, personal traits). On this basis, the researchers reintegrated and reconstructed the first-level codes according to their intrinsic connections and logical hierarchy, linking the scattered codes into three more general second-level codes: occupational gender expectations, work competence, and personal traits required for work. Finally, the core concepts in the data were identified and integrated to construct and refine the theoretical framework, ultimately yielding three third-level codes: competence-based occupational gender stereotypes, trait-based occupational stereotypes, and occupational gender expectations. In addition, during the interviews, we noticed differences in how respondents who were familiar with civil aviation occupations and those who were not expressed their views. Therefore, “familiarity” may also be a variable that warrants separate attention.

The results revealed that gender stereotypes in aviation professions exhibited pronounced gender-based and skill/service-oriented dichotomies. For instance, pilots were predominantly perceived as a high-expertise, men-dominated occupation, whereas flight attendants were frequently labeled as a feminized role requiring lower professional competence.

Expert Review and Research Group Discussion. Two experts – one psychometrist and one sexologist – provided their feedback regarding the wording and response alternatives of the items. A research group discussion was conducted with eight individuals with backgrounds in psychology and research methodology. Subsequently, we merged items with similar meanings and eliminated those with improper expressions or ambiguous meanings. Finally, we selected 15 items from the pool.

Pre-test, Final Expert Review, and Research Group Discussion. We administered a pre-test, followed by a final expert review and research group discussion, to check whether the items were easily understandable and unambiguous and to modify any inappropriate items.

After minor revisions during the pre-test and final review, the 15-item scale was retained. All items were rated on a 7-point scale. These 15 items consisted of five items on occupational gender competence, five on occupational gender personality traits, and five on gender inference. Furthermore, we reverse-scored several items to avoid acquiescent response patterns. Finally, we anonymized responses and stored them as digital codes to mitigate social desirability bias. In addition, we added five 7-point items assessing familiarity with each of the five civil aviation occupations (e.g., “How familiar are you with the aviation security officer profession?”), to serve as auxiliary analysis variables for subsequent research.

### Study 1b: item selection and factor structure exploration

#### Methods

***Participants.*** We recruited participants using a dual-channel approach as follows: (a) online surveys distributed through the Credamo platform (a relatively mature and reliable questionnaire data collection platform in China that is widely utilized by researchers) and the WeChat app, and (b) on-site questionnaire administration at civil aviation universities.

We excluded sample data that failed attention checks (e.g., “Please select ‘Very familiar’ for this item”) or the logic-check item (i.e., participants were asked to re-select their gender at the end of the questionnaire), as well as outliers in response time (using 45.63s – the mean response time of three research team members familiar with the questionnaire as the lower limit; responses below this time were removed). In addition, because all questions were set as mandatory, there were no missing values for any item. A total of 707 participants’ data were utilized in Study 1b, yielding a valid response rate of 97.12%. Participants’ ages ranged from 17 to 28. The sample size was greater than 300, and the ratio of the sample size to the number of variables was greater than 10:1, suggesting an appropriate sample size (Liu, 2019). Table 1 presents the participants’ detailed demographic characteristics.

**TABLE 1:** Participants’ demographic characteristics in Study 1b.

Variable	Second stage
	(*N* = 707)
Gender, N (%)	
Man	382 (54.0%)
Woman	325 (46.0%)
Major, N (%)	
Flight technology	160 (22.6%)
Air traffic control	60 (8.5%)
Aviation security	45 (6.4%)
Aircraft maintenance	70 (9.9%)
Flight attendant	230 (32.5%)
Other	142 (20.1%)
Hometown, N (%)	
City	424 (60.0%)
Town	103 (14.6%)
Countryside	180 (25.4%)

***Measures.*** The initial scale comprised 15 items, with participants responding to each item using a 7-point Likert scale. After reverse-scoring several items, the highest possible total score was 105, with higher scores indicating higher levels of gender stereotypes in civil aviation occupations. The initial scale was developed and tested in Chinese. In addition, the questionnaire also included five demographic items, one attention check item, one logic-check item and five items assessing familiarity with five civil aviation occupations.

***Data analysis.*** We conducted all analyses using SPSS 22.0 and Mplus 8.0. We utilized the following exclusion criteria for item selection. (1) *t*-test: We compared the items between groups with the top 27% and bottom 27% of total scores. These items’ scores did not differ significantly between groups (*p* > 0.05). (2) Responsiveness analysis method: We identified items that had over two alternatives with a selection rate below 10%. (3) Correlation coefficient method: We identified items whose correlations with the scale’s total score were below 0.6. (4) Principal component analysis (PCA) with varimax rotation: We extracted a single component and removed items with loadings lower than 0.4. This PCA was used only for preliminary item screening to ensure each item was sufficiently unidimensional; it was not intended to determine the final factor structure. We eliminated items that fulfilled even one of these four criteria.

#### Results

**Item selection.** Only one item was removed in each item selection round. Based on the above four item-selection criteria, five items were removed from the 15 items entering this stage, yielding a final 10-item scale. The five removed items were all personality-related items concerning civil aviation occupations (e.g., “Male temperament is more appropriate for air traffic control”). These items exhibited cross-loadings or high collinearity with the competence items, and therefore failed the PCA-based screening criterion.

**Exploratory factor analysis (EFA).** After item screening, we performed EFA on the final 10 items. We first performed the Kaiser–Meyer–Olkin (KMO) test, Bartlett’s sphericity test, and item correlation analysis. The results indicated that most correlation coefficients exceeded 0.3 (*p* < 0.001), the KMO test value was 0.854 (>0.5), and the measure of sampling adequacy for each item exceeded 0.5 on the diagonal of the image correlation matrix. Therefore, the data fulfilled the prerequisites for EFA.

We conducted EFA on the final 10 items using Mplus 8.0 with the WLSMV estimator and GEOMIN oblique rotation. Because the items were measured on a Likert scale and the factors were expected to correlate, an oblique rotation was appropriate. We extracted one to three factors. The model fit indices for each solution are presented in Tables 2, 3.

**TABLE 2:** Model comparisons.

Model	*k*	χ ^2^	*df*	*p*
1-factor	10	2052.61	35	<0.001
2-factor	19	867.57	26	<0.001
3-factor	27	455.21	18	<0.001
**Model comparisons**		**χ ^2^**	***df***	***p***
1-factor against 2-factor		978.62	9	<0.001
2-factor against 3-factor		399.84	8	<0.001

k refers to the number of parameters.

**TABLE 3:** Model fit indices for exploratory factor analysis models.

Model	TLI	CFI	RMSEA (90% *CI*)	SRMR
1-factor	0.826	0.865	0.286 [0.275, 0.296]	0.178
2-factor	0.902	0.944	0.214 [0.202, 0.226]	0.043
3-factor	0.927	0.977	0.185 [0.171, 0.200]	0.029

The eigenvalues for the sample correlation matrix were 5.677, 1.824, 0.671, and below. Only the first two factors had eigenvalues greater than 1.0, and the scree plot showed a clear elbow after the second factor, supporting a two-factor solution. The two factors together accounted for 73.12% of the variance.

Although the three-factor model showed slightly better fit indices, we did not retain it for two reasons. First, the standardized loading of X2 on Factor 1 exceeded 1.0 (1.027), indicating model misspecification and over-extraction. Second, the third factor did not correspond to any theoretically meaningful construct, as it primarily captured idiosyncratic variance from a small subset of items rather than a coherent substantive dimension.

By contrast, the two-factor model yielded all factor loadings within the proper range (0.525–0.924; Table 4), with no estimation problems, and aligned with our theoretically proposed structure. The two factors were moderately correlated (r = 0.546), suggesting they are related but distinct constructs. Although the RMSEA for the two-factor model was 0.214, which is above the conventional cutoff of 0.08, the other fit indices indicated acceptable fit (CFI = 0.944, TLI = 0.902, SRMR = 0.043). Given that this measurement model has relatively low degrees of freedom, prior research has cautioned that RMSEA can be unreliable and may over-reject correctly specified models in such contexts (Martinko et al., 2018; Shi et al., 2021). We therefore prioritized SRMR and CFI/TLI in evaluating model fit. Thus, based on the eigenvalue criterion, model interpretability, theoretical consistency, and the convergent evidence from multiple fit indices, we selected the two-factor model.

**TABLE 4:** Items and factor loadings of the two-factor model.

Item	Factor 1	Factor 2
Q1. Males are more competent than women in piloting aircraft	0.886	
Q2. Males possess superior abilities for air traffic control	0.888	
Q3. Males are physically more capable as aviation security officers	0.924	
Q4. Males have stronger technical aptitude for aircraft maintenance	0.914	
Q5. Females demonstrate better service skills as flight attendants	0.695	
Q6. Pilots are more likely to be male		0.883
Q7. Air traffic controllers are more likely to be male		0.847
Q8. Aviation securities are more likely to be male		0.880
Q9. Aircraft maintenance technicians are more likely to be male		0.782
Q10. Flight attendants are more likely to be female		0.525

The five items comprising Factor 1 had loadings ranging from 0.695 to 0.914, while the five items comprising Factor 2 had loadings ranging from 0.525 to 0.883 (Table 4).

### Study 1c: reliability and validity

#### Methods

Participants. We recruited participants through the Credamo platform, the WeChat app, and civil aviation universities. After data cleaning (using the same criteria as in Study 1b), we retained 1,465 participants’ valid data, with a valid response rate of 97.54%. Participants’ ages ranged from 17 to 28. The sample size was greater than 300, while the ratio of the sample size to the number of variables was greater than 10:1, suggesting an appropriate sample size (Liu, 2019). Table 5 presents participants’ detailed demographic characteristics.

**TABLE 5:** Participants’ demographic characteristics in Study 1c.

Variable	Third stage
	(*N* = 1,465)
Gender, N (%)	
Man	667 (45.5%)
Woman	798 (54.5%)
Major, N (%)	
Flight technology	260 (17.7%)
Air traffic control	107 (7.3%)
Aviation security	67 (4.6%)
Aircraft maintenance	114 (7.8%)
Flight attendant	376 (25.7%)
Other	541 (36.9%)
Hometown, N (%)	
City	857 (58.5%)
Town	246 (16.8%)
Countryside	362 (24.7%)

***Measures.*** We employed the CA-OGSS comprising 10 items (five items for Factors 1 and 2, respectively). Participants responded to each item using a 7-point Likert scale. After reverse-scoring several items, the highest possible total score was 70, with higher scores indicating higher levels of gender stereotypes in civil aviation occupations. The CA-OGSS was tested in Chinese. In addition, the questionnaire also included five demographic items, one attention check item, one logic-check item, five items assessing familiarity with five civil aviation occupations, and the five items that were removed in Study 1b.

***Data analysis***. We assessed the scale’s construct validity using confirmatory factor analysis (CFA). The reliability tests included Pearson correlation coefficients between item and total scores, Cronbach’s α, and split-half reliability. We conducted all analyses using SPSS 22.0 and Mplus 8.0.

#### Results

***Descriptive statistics.*** The absolute value of each item’s skewness coefficient ranges between 0.032 and 0.782, while the kurtosis coefficient’s absolute value ranges between 0.054 and 0.681. Table 6 displays all items’ means and standard deviations.

**TABLE 6:** All items’ descriptive statistics.

Factor	Item	*M*	*SD*
*Gender-based competence*	Q1	4.74	1.479
	Q2	4.45	1.451
	Q3	5.11	1.526
	Q4	4.92	1.515
	Q5	5.16	1.413
*Gender stereotype inference*	Q6	5.04	1.239
	Q7	4.55	1.123
	Q8	5.15	1.297
	Q9	4.89	1.339
	Q10	5.07	1.271

***Construct validity.*** We conducted a CFA on the two-factor model comprising two latent variables and 10 measurement indicators, with a total of 55 data points (p*(p+1)/2). There are 21 parameters to estimate, including 10 factor loadings, one covariance between the two factors, and an error variance of 10 items [55–21 = 34 (*df*)]. Each factor has more than three items without relevant errors, implying that the model conforms to the rules of CFA identification.

The two-factor model showed acceptable fit based on convergent evidence: root mean square error of approximation (RMSEA; 90% confidence interval [*CI*]) = 0.072 (0.062, 0.084); comparative fit index (CFI) = 0.995; Tucker–Lewis index (TLI) = 0.989; and standardized root mean squared residual (SRMR) = 0.013, and no modification indices above 20 were observed. Although the χ^2^/*df* value was 6.77, which exceeded the conventional cutoff of 5. However, χ^2^/*df* is highly sensitive to sample size and model complexity, and with a relatively large sample, it can be inflated even when the model is approximately correct (Brown, 2015). In contrast, CFI, TLI, RMSEA and SRMR all indicated good fit. Taken together, these results support the construct validity of the final 10-item CA-OGSS.

The standardized factor loadings for Factor 1 ranged between 0.718 and 0.918, while those for Factor 2 ranged between 0.639 and 0.847 (Figure 1).

**FIGURE 1:**
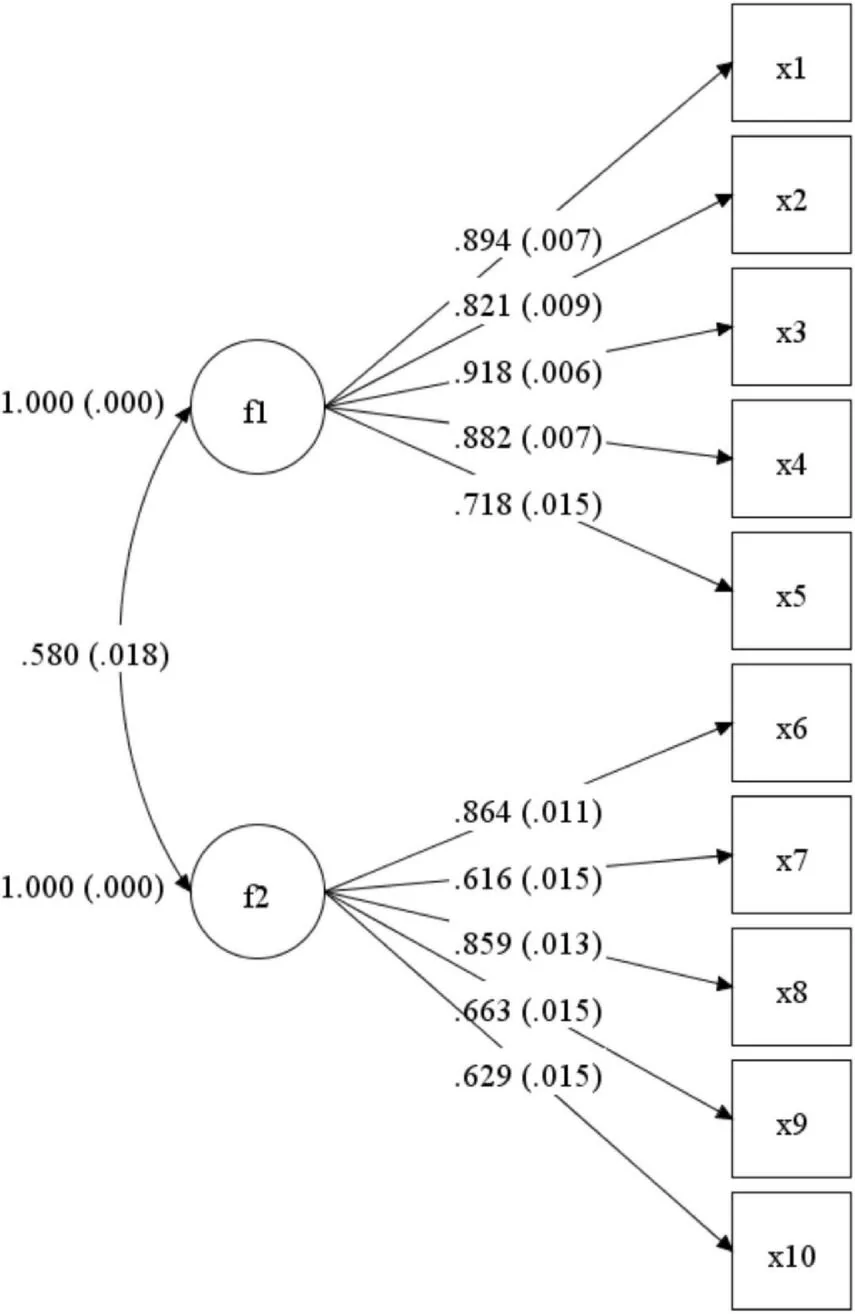
Path diagram of confirmatory factor analysis.

To distinguish whether Factor 2 (*Gender stereotype inference*) was based on participants’ observations of the actual gender proportions in civil aviation occupations or on gender stereotypes, we conducted a correlation analysis between the total score of Factor 2 and the total score of five items assessing familiarity with five civil aviation occupations. These five familiarity items were rated on a 7-point scale, with higher scores indicating greater familiarity. Pearson correlation analysis showed a significant but weak positive correlation between Factor 2 and familiarity (*r* = 0.101, *p* = 0.001).

Gender Measurement Invariance Testing. To test whether the CA-OGSS satisfies measurement invariance between men and women, nested models were compared using the DIFFTEST procedure (Li and Liu, 2011). The single-group confirmatory factor analysis (CFA) results are presented in Table 7. The scale showed good model fit in both groups (men and women), and the single-group models can serve as baseline models for subsequent measurement invariance testing.

**TABLE 7:** Single-group CFA results for the CA-OGSS.

Group	χ ^2^	*df*	TLI	CFI	RMSEA (90% *CI*)	SRMR
Men (*N* = 667)	142.62	34	0.984	0.993	0.078 [0.061, 0.095]	0.020
Women (*N* = 798)	150.28	34	0.986	0.993	0.077 [0.061, 0.093]	0.015

Using the men as the reference group, gender measurement invariance was tested. First, configural invariance was examined. The results indicated that the scale achieved configural invariance across men and women (χ^2^/df = 4.31, CFI = 0.991, TLI = 0.982, RMSEA [90% CI] = 0.076 [0.069, 0.087]). However, when testing weak invariance, the model fit deteriorated significantly (χ^2^/df = 21.88, CFI = 0.971, TLI = 0.954, RMSEA [90% CI] = 0.136 [0.126, 0.145]; DIFFTEST Δχ^2^ (Ma, 2000) = 306.31, *p* < 0.001), indicating that weak invariance was not supported across genders. Therefore, the scale only satisfied configural invariance, but not weak invariance, between men and women. That is, although the factor structure was identical across the two groups, at least some item factor loadings differed between groups, meaning that the relationships between items and the latent construct (i.e., measurement units) were not consistent across genders. Based on these results, subsequent tests of strong invariance (scalar invariance) and strict invariance were not continued, and direct comparisons of latent means or observed total scores between men and women should not be conducted.

***Reliability analysis.*** The Pearson correlation coefficients between each item and the total scores were between 0.564 and 0.898. The total scores on Factor 1 and its five constituent items ranged between 0.620 and 0.918, while the total scores on Factor 2 and its five constituent items ranged between 0.561 and 0.878. The *Cronbach’s α* values for the full scale, Factor 1, and Factor 2 were 0.884, 0.910, and 0.839, respectively. The scale’s split-half reliability was 0.756. Collectively, these results suggest that the CA-OGSS exhibits acceptable reliability. The complete CA-OGSS (Chinese and English versions) is provided in Supplementary Appendix A.

### Study 2: scale application – influence of gender stereotypes in aviation professions

#### Methods

***Participants.*** We recruited college students majoring in aviation-related fields from five civil aviation universities. Considering the large number of items, we included three attention check items (e.g., “Please calculate 5–3 = ?”) and one logic-check item (i.e., participants were asked to re-select their gender). We excluded sample data that failed attention checks and outliers in response time (using 1 min 39.98 s – the mean response time of three research team members familiar with the questionnaire – as the lower limit; responses below this time were removed). After data cleaning, a total of 830 valid participants were included, yielding a valid response rate of 90.12% (*N*_*_Women*_ = 346 [41.70%]), with the following distribution across majors: 226, 86, 65, 98, and 345 students in flight technology, air traffic control, aviation security, aircraft maintenance engineering, and flight attendant training, respectively. Participants’ ages ranged from 17 to 28. The sample size was greater than 300, while the ratio of the sample size to the number of variables was greater than 10:1, indicating an appropriate sample size (Liu, 2019).

#### Measures

##### Gender stereotypes in civil aviation professions

Gender stereotypes in civil aviation professions were measured using the CA-OGSS scale, which was developed in Study 1. The final measure comprised 10 items (five and five for Factors 1 and 2, respectively). Participants responded to each item using a 7-point Likert scale. Higher scores indicated stronger gender stereotypes in civil aviation. The scale exhibited acceptable internal consistency: The Cronbach’s α coefficients for the total scale, Factor 1, and Factor 2 were 0.891, 0.918, and 0.872, respectively.

##### Professional identity

Professional identity was measured using a questionnaire assessing college students’ professional identity developed by Qin (Qin, 2009). The scale comprised 23 items. All questionnaire items employ a 5-point Likert scale, with 1 representing “Completely inconsistent” and 5 representing “Completely consistent.” A higher score indicates a higher level of professional identity. In this study, the *Cronbach’s α* coefficient of the adjusted Professional Identity Scale was 0.977, indicating satisfactory reliability and suitability for administration.

##### Self-efficacy

This study adopted the General Self-Efficacy Scale developed by Schwarzer et al. (1997). The scale consists of 10 items with a 7-point Likert scale for scoring, where a higher score indicates a higher level of self-efficacy. In this study, the *Cronbach’s α* coefficient of the scale was 0.978.

In addition to the aforementioned scale, the questionnaire also included five demographic items, one logic-check item and three attention check items.

#### Data analysis

After excluding data with excessively short response times and those that failed attention check items, 16.02% of the participants had a missing value on one item. Therefore, we imputed the missing value using the mean of the questionnaire to which that item belonged. In the data processing stage, we conducted the following analyses: First, as all study data were collected through self-report, issues of common method bias (CMB) are possible. Therefore, this study employed Harman’s single-factor test and the unmeasured latent method construct (ULMC) approach to assess the severity of CMB. Subsequently, descriptive statistics and correlation analyses were conducted to assess gender stereotypes in civil aviation professions, professional identity, and self-efficacy. Finally, to investigate the moderated mediation model, mediation analysis was performed using the PROCESS macro in SPSS 29.0, and two regression equation models were constructed (Hayes, 2017). The bootstrap method was applied to further examine significant relationships among these variables, with 5,000 resamples and a 95% *CI*. We performed all statistical analyses using SPSS 29.0 and Mplus 8.3.

#### Results

***Tests for CMB.*** To examine CMB, we first conducted Harman’s single-factor test. The EFA results indicated that the first factor extracted without rotation accounted for 43.15% of the total variance; although it exceeded the stricter 40% threshold, it remained below the conventional 50% cutoff. Subsequently, we further conducted the ULMC approach. First, the fit indices of the theoretical factor model were as follows:(*χ^2^/df* = 9.26; CFI = 0.962 and TLI = 0.960; RMSEA = 0.100 [90% *CI*: 0.098, 0.102]; and SRMR = 0.055). Next, the fit indices of the model with the method factor added were as follows: (*χ^2^/df* = 7.21; CFI = 0.973 and TLI = 0.970; RMSEA = 0.086 [90% *CI*: 0.084, 0.089]; and SRMR = 0.025). The method factor accounted for an average of 49.96% of the indicator variance. Despite this, adding the method factor did not substantially improve model fit (ΔCFI = 0.011, ΔTLI = 0.010, ΔRMSEA = -0.014, ΔSRMR = -0.030), suggesting that common method bias did not seriously distort the relationships among the theoretical constructs. Overall, these results suggest that common method bias was not a serious concern in this study.

***Descriptive statistics and correlation analysis.*** Table 8 presents the descriptive statistics and correlations among the study variables. The results indicated that gender stereotypes in civil aviation professions were significantly positively correlated with professional identity (*r* = 0.370, *p* < 0.01), significantly positively correlated with self-efficacy (*r* = 0.372, *p* < 0.01), and significantly positively correlated with gender (*r* = 0.446, *p* < 0.01). Additionally, professional identity was significantly positively correlated with self-efficacy (*r* = 0.756, *p* < 0.01) and significantly positively correlated with gender (*r* = 0.136, *p* < 0.01). Moreover, self-efficacy was significantly positively correlated with gender (*r* = 0.184, *p* < 0.01).

**TABLE 8:** Means, standard deviations, and correlations of study variables.

Variable	1	2	3	4
Gender stereotypes in civil aviation professions	–	–	–	–
Professional identity	0.370**			
Self-efficacy	0.372**	0.756**		
Gender	0.446**	0.136**	0.184**	
M ± SD	4.89 ± 1.00	3.90 ± 0.79	5.07 ± 1.14	1.58 ± 0.49

(1) Means and standard deviations were computed as the total scale score divided by the number of items. (2) Correlation analyses were conducted using Spearman’s rank-order correlation. (3)

**p < 0.01.

***Mediation analysis.*** Table 9 presents professional identity’s mediating effect on the relationship between gender stereotypes in civil aviation occupations and self-efficacy. The indirect effect was 0.312, accounting for 81.3% of the total effect, with a 95% bootstrap *CI* of [0.244, 0.385]. The confidence interval does not contain 0, indicating that this mediating effect is significant. Additionally, the direct effect was 0.072 (95% *CI* [0.019, 0.125]), which remained significant, suggesting a partial mediation.

**TABLE 9:** Testing for mediation effects of professional identity.

Mediating variable	Effect	Effect value	*SE*	*95% CI*
Professional identity	Total effect	0.384	0.037	[0.311, 0.457]
	Direct effect	0.072	0.027	[0.019, 0.125]
	Indirect effect	0.312	0.036	[0.244, 0.385]

Table 10 presents the moderated mediation model’s effects. In Equation 1, gender stereotypes in civil aviation occupations did not significantly predict professional identity (*B* = 0.036, *SE* = 0.106, 95% *CI* [-0.171, 0.243]), while the interaction term between gender stereotypes and gender significantly predicted professional identity (*B* = 0.162, *SE* = 0.061, 95% *CI* [0.043, 0.281]), indicating that gender moderated the first stage of the mediation. In Equation 2, professional identity significantly predicted self-efficacy (*B* = 1.064, *SE* = 0.034, 95% *CI* [0.998, 1.131]). Additionally, the main effect of gender stereotypes was significantly negative (*B* = −0.464, *SE* = 0.102, 95% *CI* [−0.665, −0.263]), and the interaction between gender stereotypes and gender significantly predicted self-efficacy (*B* = 0.304, *SE* = 0.059, 95% *CI* [0.188, 0.420]). We further tested whether gender moderated the path from professional identity to self-efficacy by adding the M x W interaction term. The M x W interaction was non-significant (*B* = 0.131, *SE* = 0.032, 95% *CI* [−0.001, 0.263]), indicating that gender did not moderate the association between professional identity and self-efficacy. Figure 2 illustrates the final moderated mediation model.

**TABLE 10:** Testing for moderated mediation effects.

Variables and model fit	Equation 1			Equation 2			Equation 3		
	(Professional Identity, M)			(Self-Efficacy, Y)			(Self-Efficacy, Y)		
	*B*	*SE*	*95% CI*	*B*	*SE*	*95% CI*	*B*	*SE*	*95% CI*
Independent variables									
X	0.036	0.106	[-0.171, 0.243]	-0.464***	0.102	[-0.665, -0.263]	0.034	0.017	[−0.025, 0.093]
W	−0.831**	0.289	[-1.398, -0.264]	-1.278***	0.281	[-1.830, -0.726]	−0.358	0.568	[−0.873, 0.156]
X × W	0.162**	0.061	[0.043, 0.281]	0.304***	0.059	[0.188, 0.420]	–	–	–
M	–	–	–	1.064***	0.034	[0.998, 1.131]	0.868***	0.016	[0.643, 1.092]
M × W	–	–	–	–	–	–	0.131	0.032	[−0.001, 0.263]
Model fit									
*R* ^2^	0.143			0.613			0.602		
*F*	45.944***			326.021***			311.841***		

(1) B: unstandardized regression coefficient; SE: standard error. (2) X = Gender Stereotypes in Civil Aviation Professions; Y = Self-Efficacy; M = Professional Identity; W = gender (1 = woman, 2 = man). (3)

***p < 0.001,

**p < 0.01. (4) The results for Equations 1 and 2 were obtained using Model 8 in SPSS PROCESS, and Equation 3 was obtained using Model 14.

**FIGURE 2:**
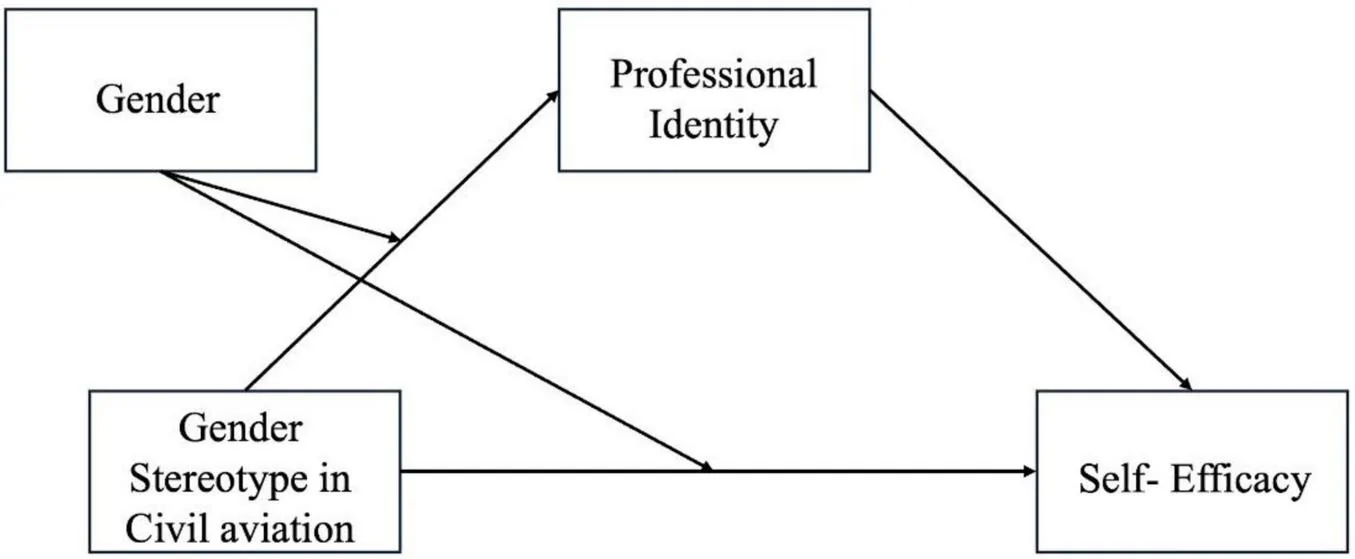
Graph of the Moderated mediation model.

It is worth noting that although the direct effect of X on Y was positive in Table 9 (0.072), the coefficient of X on Y became significantly negative in Equation 2 of Table 10 (−0.464). This shift occurs because the inclusion of the interaction term (X*W) changes the interpretation of X’s coefficient. Specifically, in a moderated mediation model, the coefficient for X no longer represents the overall main effect, but rather the conditional effect of X on Y when the moderator (gender) is at a specific reference value.

A simple slope analysis of the moderating effect on the direct path revealed a significant gender difference in the direct effect’s moderation: Among women (W = 1), gender stereotypes in civil aviation professions exhibited a significant negative predictive effect on self-efficacy (*B*__simple_ = -0.160, 95% *CI* = [−0.256, −0.064]). By contrast, among men (W = 2), gender stereotypes in civil aviation professions demonstrated a significant positive predictive effect on self-efficacy (*B*__simple_ = 0.144, 95% *CI* = [0.074, 0.214]).

Furthermore, a bootstrap conditional indirect effect analysis indicated that the path X → M was significant and moderated by W (*B* = 0.162, *p* = 0.008). The indirect effect differed significantly between men and women (Index = 0.172, 95% *CI* [0.007, 0.316]): Specifically, it was significant among women (*B* = 0.210, 95% *CI* [0.087, 0.353]); by contrast, the indirect effect was stronger and also significant among men (*B* = 0.383, 95% *CI* [0.291, 0.474]). These results suggest that the mediating pathway “gender stereotypes in civil aviation professions → professional identity → self-efficacy” is significantly stronger among men than among women, and this difference is significant.

#### Discussion

Based on previous theories of gender stereotypes and the SCM, this research examines the structure of occupational gender stereotypes in the civil aviation industry in mainland China and develops the CA-OGSS, which demonstrates acceptable reliability and validity in the Chinese cultural context. Further, using the CA-OGSS, the research examined the associations between gender stereotypes in civil aviation professions on the self-efficacy of university students majoring in civil aviation. The findings indicate that these stereotypes were associated with students’ self-efficacy through professional identity’s mediating role. Moreover, gender moderated both the direct path and the first half of the mediation pathway, suggesting a moderated mediation model. However, given the cross-sectional design, these findings should be interpreted as correlational rather than causal.

Previous studies on occupational gender stereotypes have predominantly assessed the public’s perception of the congruence between occupations and gender, with limited focus on specific industries (e.g., civil aviation) and a tendency to depend on unidimensional evaluation frameworks. Jiang et al. (2010) identified 10 male-typed and 7 female-typed occupations based on a survey and the Ministry of Labor’s occupational classification system, employing attitude statements such as “Men are more suitable for the job of a doctor” to assess university students’ occupational stereotypes. A key limitation of this approach is that the resulting data do not readily enable differentiation between “gender stereotypes” and the “recognition of existing industry demographics” during interpretation. In contrast, the present study’s Factor 2 (Gender stereotype inference) dimension employed familiarity with civil aviation occupations to distinguish whether participants’ responses were based on stereotypes or on observations of the actual gender composition of these occupations; this approach enhanced the scale’s construct validity to some extent. Meanwhile, Factor 1 (Gender-based competence) integrated occupational stereotypes and gender stereotypes within the SCM. This multidimensional framework broadens the scope of occupational stereotype measurement and emphasizes typical roles in the civil aviation industry, thereby facilitating a more nuanced and comprehensive analysis of the structure of gender stereotypes in this field.

The present research developed the CA-OGSS through a comprehensive process involving a literature review, interviews, item pool generation, and item screening, followed by an evaluation of its reliability and validity. The results indicate that the scale comprises the following two factors: “Gender-based competence” (Factor 1) and “Gender stereotype inference” (Factor 2). Factor 1 – based on the SCM – assesses perceptions of civil aviation occupations and gender across dimensions of competence and warmth, thereby identifying whether gender biases associated with a given occupation stem from perceived advantages linked to gender-based competence or warmth. Factor 2 draws on the item-construction methods employed in the *Bem Sex-Role Inventory* (Bem, 1974) and *The Structure and Development of Gender Stereotypes in Adolescents and Children* (Liu, 2007). It utilizes typical civil aviation professions as referents to assess the perceived congruence between gender and these occupations. It should be noted that Factor 2 may partly reflect awareness of actual gender distributions in these occupations rather than pure stereotype endorsement. To address this concern, we examined the correlation between Factor 2 and familiarity with the five civil aviation occupations. The correlation was significant but weak, suggesting that Factor 2 is not merely a reflection of familiarity with real-world gender proportions. Nevertheless, future research should further disentangle stereotype-based inferences from accurate perceptions of occupational gender composition.

Extant studies on occupational and gender stereotypes have reported a cognitive tendency to link “gender – traits – profession,” a pattern that is also evident in Chinese cultural contexts. Specifically, men-dominated industries typically exhibit characteristics that exclude women, as reflected in evaluations such as “a woman’s view is not worth considering [妇人之见，不足与谋]” and “womanly compassion and indecisiveness [妇人之仁，优柔寡断].” These descriptors not only undermine women’s capabilities but also emphasize the perceived misalignment between femininity and the demands of high-intensity, men-dominated fields (Daulet et al., 2019; Gümüş, 2021). On the contrary, women-dominated industries frequently diminish the status of their men workers, who face traditional prejudices such as being “overqualified yet underachieving [大材小用]” or “a man without masculinity is like iron that never becomes steel [男无性铁无钢].” Such discrimination is likewise grounded in judgments concerning perceived competence and personality (Ayub and Yasin, 2022). The two-factor model of the CA-OGSS developed herein showed acceptable fit. Given that χ^2^/*df* is highly sensitive to sample size, these results should be interpreted with caution. Overall, the findings suggest that gender stereotypes associated with civil aviation occupations in Chinese society also encompass the dimensions of gender-based competence and gender stereotype inference.

Previous studies have established that occupational gender stereotypes significantly impact individuals’ career development. Wang and Yu (2019) surveyed men nursing students and found that stronger occupational gender stereotypes are associated with lower professional identity, with higher levels of stereotyping correlating with a reduced sense of belonging and commitment to the field. Similar patterns have been documented in other men-dominated domains, such as the military and STEM, wherein a lack of women role models reinforces the perception that “this field is not for women,” thereby undermining women’s professional identity, which, in turn, reportedly decreases self-efficacy (Smith and Rosenstein, 2017). Likewise, the civil aviation industry exhibits pronounced gender segregation across roles, such as men pilots and women flight attendants (Casebolt, 2023; Williams, 2003). A moderated mediation model was developed to further validate the utility of the CA-OGSS and examine the associations among gender stereotypes, professional identity, and self-efficacy in civil aviation.

Path analysis indicated that gender stereotypes in civil aviation occupations were associated with self-efficacy through professional identity’s mediating role, demonstrating a significant mediation effect. This finding aligns with existing research conducted in military contexts (Smith and Rosenstein, 2017). From the perspective of social identity theory (Tajfel and Turner, 2004), individuals gain positive self-esteem and a sense of self-worth by identifying with a social group, such as “aviation professionals.” Thus, stereotypes associated with civil aviation may be internalized by students as positive group attributes, thereby reinforcing social identity and, in turn, potentially enhancing self-efficacy.

Additionally, this research identified gender as a significant moderator in this framework, affecting both direct and indirect pathways. In the direct effect, gender played a bidirectional moderating role: For men students, gender stereotypes positively predicted self-efficacy, whereas for women students, these stereotypes negatively predicted self-efficacy. This disparity may be attributable to the aviation industry’s men-dominated environment, wherein men may perceive their gender as an inherent advantage, thereby boosting their professional confidence and self-affirmation. By contrast, women may internalize stereotypes directing them toward service-oriented roles, such as flight attendants. Such roles are often socially devalued – particularly in East Asian cultural settings – which can diminish self-efficacy (Chang and Chen, 2019; Zhang et al., 2016). Furthermore, women are more susceptible to stereotype threat (Steele and Aronson, 1995), further compromising their self-efficacy.

Regarding the indirect effect, the reinforcing association of stereotypes on professional identity was more pronounced among men than among women. This finding aligns with those of Wang and Yu (2023) and Clark et al. (2021), who reported that, owing to the pervasive perception that men are more suited to STEM fields than women, men students generally develop a stronger self-concept in these domains and exhibit greater motivation and academic confidence than women. This pattern also aligns with the expectations of role congruity theory (Eagly and Karau, 2002). Traditional stereotypes associated with core positions in civil aviation, such as pilots and aircraft maintenance technicians, are highly congruent with male gender roles. Consequently, when men students encounter these stereotypes, they may experience a stronger sense of role belonging, which may enhance their professional identity. However, for women students, such stereotypes may conflict with their gender identity, thereby exerting a weaker improvement effect on their sense of professional identification.

It is important to note that the present study only established configural invariance, but not weak invariance, across gender. Therefore, direct comparisons of latent means or total scores between men and women should not be conducted. The observed differences in the associations between stereotypes and outcomes for men and women should be interpreted with caution, as they may partly reflect measurement non-equivalence rather than true group differences.

The observed disparity in the influence of occupational gender stereotypes between men and women reflects the theory of hegemonic masculinity (Messerschmidt, 2012), which posits that femininity is often socially devalued, whereas masculinity is socially esteemed. Central to gender stereotypes is the normative expectation that men and women should embody masculine and feminine traits, respectively (Jackson and Bussey, 2023). Consequently, men who strongly exhibit such stereotypes are likely to affirm masculinity – a socially rewarded attribute – thereby potentially developing higher self-efficacy. By contrast, women with high stereotype adherence usually internalize femininity, which is frequently stigmatized, thus potentially lowering self-efficacy. Furthermore, Jackson and Bussey (2020) study on gender identity differences between men and women found that men experience greater pressure to conform to gender norms and frequently avoid traits associated with other genders, whereas women report lower internal pressure to align with their gender role but exhibit greater compliance with cross-gender expectations.

### Theoretical contributions and practical implications

This research developed the CA-OGSS, thus providing an empirical reference for future work on gender stereotypes in civil aviation and other professional fields. Further, it reveals how gender stereotypes may function as a “double-edged sword” in men-dominated environments, such as the civil aviation industry: Despite enhancing the group’s collective identity, they may also inadvertently marginalize women members and undermine their self-belief. This insight offers a novel perspective for understanding gender dynamics in the occupational culture of specific industries in China.

This research holds significant implications for talent development and team construction in China’s civil aviation sector. To achieve talent optimization and gender equality, consciously deconstructing the traditional stereotype that “men are more suited” for certain roles – both in professional competency education and within the workplace – is crucial. Increasing the visibility of female role models, such as accomplished female pilots and aircraft maintenance engineers, can provide positive reference points for aviation students. Eliminating gender-based competency evaluations and focusing solely on professional capability can help students develop a competence-centered sense of professional identity and self-efficacy.

### Limitations and future directions

This research exhibits several limitations. First, the sample consisted of civil aviation students and general university students, rather than actual civil aviation employees. Although students are the future workforce, their stereotypes and experiences may differ from those of practitioners. Future research should collect data from current civil aviation employees to validate the findings in real occupational settings. Second, the use of a cross-sectional design precludes any causal inferences. Future research should adopt a longitudinal approach, collecting data at multiple critical time points to identify dynamic influences. Third, the self-report measures employed might have introduced social desirability bias. Subsequent studies should incorporate multi-method approaches – including behavioral experiments, objective indicators, and peer evaluations – to enhance the findings’ validity. Fourth, the CA-OGSS only satisfied configural invariance across gender, not weak invariance. This means that the scale cannot be used to compare latent means or total scores between men and women, and any observed gender differences in stereotype levels or associations should be interpreted with caution. Future research should further refine the scale to achieve at least weak invariance. Furthermore, future investigations should compare differences in occupational gender stereotypes between current civil aviation employees and trainees (i.e., aviation college students) and examine how these stereotypes vary among practitioners with varying experience levels.

## Data Availability

The raw data supporting the conclusions of this article will be made available by the authors, without undue reservation.
