# Supplementary material for: Development and validation of the civil aviation occupational gender stereotype scale and its impact on professional identity and self-efficacy among chinese civil aviation majors
Source: Front Psychol. 2026 Sep 30;17:1718534. doi: 10.3389/fpsyg.2026.1718534 (PMC13661356; doi:10.3389/fpsyg.2026.1718534)
Supplement: Supplementary file 1 [file Table_1.docx]

- **Appendix A**

**English Version of CA—OGSS**

| Item | Strongly Disagree | Disagree | Somewhat Disagree | Neutral | Somewhat Agree | Agree | Strongly Agree |
| --- | --- | --- | --- | --- | --- | --- | --- |
| Q1. Males are more competent than women in piloting aircraft | 1 | 2 | 3 | 4 | 5 | 6 | 7 |
| Q2. Males possess superior abilities for air traffic control | 1 | 2 | 3 | 4 | 5 | 6 | 7 |
| Q3. Males are physically more capable as aviation security officers | 1 | 2 | 3 | 4 | 5 | 6 | 7 |
| Q4. Males have stronger technical aptitude for aircraft maintenance | 1 | 2 | 3 | 4 | 5 | 6 | 7 |
| Q5. Females demonstrate better service skills as flight attendants | 1 | 2 | 3 | 4 | 5 | 6 | 7 |
| Q6.Pilots are more likely to be male | 1 | 2 | 3 | 4 | 5 | 6 | 7 |
| Q7. Air traffic controllers are more likely to be male | 1 | 2 | 3 | 4 | 5 | 6 | 7 |
| Q8. Aviation securities are more likely to be male | 1 | 2 | 3 | 4 | 5 | 6 | 7 |
| Q9. Aircraft maintenance technicians are more likely to be male | 1 | 2 | 3 | 4 | 5 | 6 | 7 |
| Q10. Flight attendants are more likely to be female | 1 | 2 | 3 | 4 | 5 | 6 | 7 |

**Chinese Version of CA—OGSS**

| 题目 | 完全不符合 | 非常不符合 | 比较不符合 | 一般 | 比较符合 | 非常符合 | 完全符合 |
| --- | --- | --- | --- | --- | --- | --- | --- |
| 1.男性在能力上更适合当飞行员 | 1 | 2 | 3 | 4 | 5 | 6 | 7 |
| 2.男性在能力上更适合当空管 | 1 | 2 | 3 | 4 | 5 | 6 | 7 |
| 3.男性在能力上更适合当空保 | 1 | 2 | 3 | 4 | 5 | 6 | 7 |
| 4.男性在能力上更适合当机务 | 1 | 2 | 3 | 4 | 5 | 6 | 7 |
| 5.女性在能力上更适合当空乘 | 1 | 2 | 3 | 4 | 5 | 6 | 7 |
| 6.飞行员更有可能是男性 | 1 | 2 | 3 | 4 | 5 | 6 | 7 |
| 7.空管更有可能是男性 | 1 | 2 | 3 | 4 | 5 | 6 | 7 |
| 8.航空安保人员更有可能是男性 | 1 | 2 | 3 | 4 | 5 | 6 | 7 |
| 9.飞机维修技术员更有可能是男性 | 1 | 2 | 3 | 4 | 5 | 6 | 7 |
| 10.空乘更有可能是女性 | 1 | 2 | 3 | 4 | 5 | 6 | 7 |
